# Bio-Functional Textiles: Combining Pharmaceutical Nanocarriers with Fibrous Materials for Innovative Dermatological Therapies

**DOI:** 10.3390/pharmaceutics11080403

**Published:** 2019-08-11

**Authors:** Daniele Massella, Monica Argenziano, Ada Ferri, Jinping Guan, Stéphane Giraud, Roberta Cavalli, Antonello A. Barresi, Fabien Salaün

**Affiliations:** 1 ENSAIT, GEMTEX—Laboratoire de Génie et Matériaux Textiles, F-59000 Lille, France; 2 Department of Applied Science and Technology, Politecnico di Torino, Corso Duca degli Abruzzi 24, 10129 Torino (TO), Italy; 3 College of Textile and Clothing Engineering, Soochow University, Suzhou 215123, China; 4 Department of Drug Science and Technology, University of Turin, Via P. Giuria 9, 10125 Torino, Italy

**Keywords:** textiles, biomedical materials, regulatory issues, skin, transdermal drug delivery, nanocarriers, electrospinning, encapsulation, fibers

## Abstract

In the field of pharmaceutical technology, significant attention has been paid on exploiting skin as a drug administration route. Considering the structural and chemical complexity of the skin barrier, many research works focused on developing an innovative way to enhance skin drug permeation. In this context, a new class of materials called bio-functional textiles has been developed. Such materials consist of the combination of advanced pharmaceutical carriers with textile materials. Therefore, they own the possibility of providing a wearable platform for continuous and controlled drug release. Notwithstanding the great potential of these materials, their large-scale application still faces some challenges. The present review provides a state-of-the-art perspective on the bio-functional textile technology analyzing the several issues involved. Firstly, the skin physiology, together with the dermatological delivery strategy, is keenly described in order to provide an overview of the problems tackled by bio-functional textiles technology. Secondly, an overview of the main dermatological nanocarriers is provided; thereafter the application of these nanomaterial to textiles is presented. Finally, the bio-functional textile technology is framed in the context of the different dermatological administration strategies; a comparative analysis that also considers how pharmaceutical regulation is conducted.

## 1. Introduction

In the last decades, research in the field of pharmaceutical technology has invested significant efforts in the development of innovative formulations for smart drug delivery [1,2]. This concept lies in the idea of administering a controlled dosage of active substance to the patient in order to be therapeutically effective while minimizing over dosage and side effects [3]. Among these innovative drug delivery technologies, the possibility of exploiting skin for pharmaceutical administration has aroused significant interest [4,5]. Indeed, skin is the largest organ of the human body and displays a significant surface area for drug administration [6]. Moreover, being the organ devoted to the internal body protection, skin can act as a barrier for harmful substances thus reducing possible side effects [7]. Therefore, skin has been considered as a possible administration route both for systemic and local drugs. According to Food and Drug Administration (FDA), the first case is defined as transdermal administration and involves the delivery of the drug through the skin layers in order to reach systemic circulation, while the latter is defined as topical administration and consists of the application of the active molecule on a particular area of the body surface [8,9]. Transdermal administration displays several advantages compared to other classic drug delivery routes such as the enteral and parenteral ones [10]. Firstly, it is noninvasive since it does not require injections. Secondly, it delivers the actives in the blood circulation avoiding the first pass metabolism in the liver [11]. Lastly, given that transdermal delivery devices can stick on the patient for several days, the number of required applications is significantly reduced; significantly improving the patient compliance to the therapy [12].

Notwithstanding the numerous advantages of the transdermal route, two main issues significantly limit its employment. In fact, the skin barrier is characterized by a great chemical complexity that limits the transport of the active substances across its numerous layers. Therefore, only a few classes of active molecules could effectively cross it to reach systemic circulation [13,14]. In order to increase the skin penetration capability of active molecules several strategies have been developed, which include use of both physical and chemical penetration enhancers [15,16,17]; however, some of these penetration enhancement strategies may result in irritating the skin in the long run [18,19]. The recent advances in nanotechnology are providing very promising solutions consisting of the development of pharmaceutical carriers that can vector the active molecules through the skin layers [20,21]. Moreover, to limit the risk of skin irritation, much attention is paid on the usage of biocompatible materials, to which skin can stand a long period contact without side effects, such as breathable textile materials [22,23,24].

In recent years, textile research has focused on development of the so-called “functional textiles” that are designed and produced not for apparel and aesthetic purpose, but for their technical properties and performances [25,26]. The application of technical textiles is broad and deals with several fields such electronics [27,28], automotive and aerospace [29,30], construction and buildings [31,32,33], protective garments [34,35] and medical textiles [36,37]. The latter application includes textiles that aim to be beneficial to the wearer’s health by owing peculiar features such as antibacterial action [38,39], protection against insects and parasites [40,41], antioxidant [42,43] and ultimately drug releasing properties [44,45]. Bio-functional textiles are produced by integrating conventional textiles and advanced pharmaceutical nanocarriers in order to provide wearable drug delivery systems, both for topical and transdermal active therapies [46,47]. Indeed, bio-functional textiles are promising products given their capability of improving dermal penetration of the active molecule and at the same time avoiding toxicity risk by employing bio compatible materials [48]. The potential societal impact of bio-functional textiles technology has catalysed a very significant interest in different scientific communities such as pharmaceutical technologists, textile engineers, materials science researchers, and chemists. Therefore, a clear understanding of the topic requires a multidisciplinary and comprehensive approach.

The present review aims to gather the most significant and recent contributions on bio-functional textiles for transdermal release and to analyze and classify them critically. The aim is to provide a set of information readily usable by scientists of different fields. Firstly, this manuscript describes the physiological nature of the dermal tissues in order to clarify the present challenges in crossing the skin barrier. After that, an overview of the mechanisms by which a substance can pass through the skin layers is provided together with the standard methodologies of penetration enhancement. Among the penetration enhancement techniques, significant attention is paid to the pharmaceutical nanocarriers inquiring the most relevant ones for dermatological applications. Furthermore, the potential of integrating the above-mentioned nanocarriers with textiles is discussed by analyzing the most common textile materials and finishing techniques. A special focus is also given on critically reviewing the different testing methodologies proposed to characterize the performance of bio-functional textiles both from the materials and the biological point of view. Finally, some critical considerations regarding the potential development of this technology are drawn, considering the regulatory issues for commercialization of these innovative products.

## 2. Nature and Physiology of the Skin

**Figure 1:**
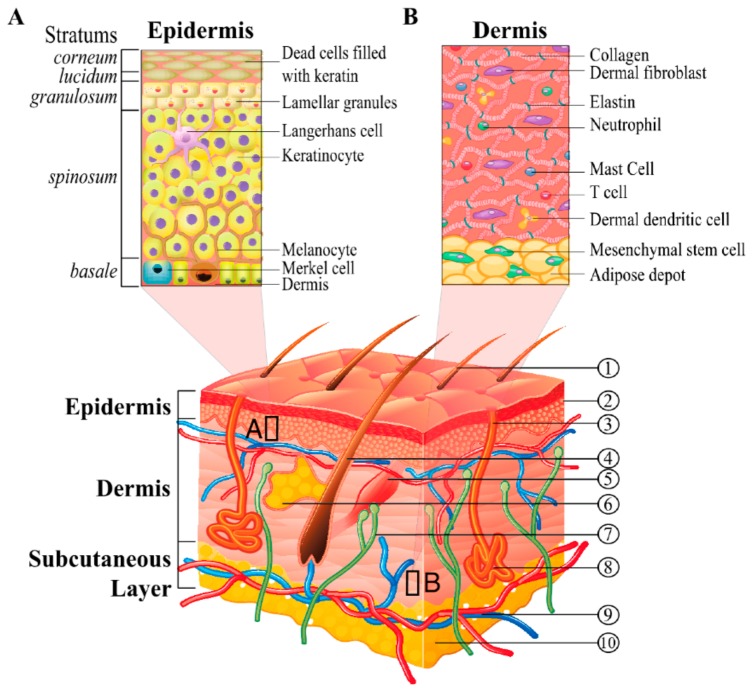
Scheme of skin layers. The references in the image point out: (1) hair shaft; (2) stratum corneum; (3) sweat-pore; (4) hair follicle; (5) arrector pili muscle; (6) sebaceous gland; (7) nerve; (8) eccrine sweat gland; (9) cutaneous vascular plexes; (10) adipose depot. Section (**A**) and (**B**) highlight a detailed structure of the epidermis and derma respectively. [Reproduced from Gaur et al. [67] which is licensed under a Creative Commons Attribution-(CC BY 4.0) International License (http://creativecommons.org/licenses/by/4.0/)].

Skin is the organ of the human body which presents the highest surface area, and it complies with essential body functions. Above all, it is a chemical and physical barrier that protects the internal tissues from the external harms such as virulent pathogens, UV light and toxic substances. Moreover, skin complies to thermal regulation, water retention, sensitive detection and self-healing [49,50]. Structurally speaking, skin consists of a series of stratified layers which are sketched and described in Figure 1. The two main skin layers are the dermis and the epidermis [51]. The epidermis is the outer skin layer and is divided into multiple layers; it is mostly constituted by keratinocytes. Keratinocytes are the most diffuse skin cells which are formed in the lowermost epidermal layer, the Stratum Basale and raise to the outermost Stratum Corneum (SC) undergoing keratinization and cellular differentiation [52]. The keratinocytes present in the SC have already undergone terminal differentiation and are therefore present as dead cells without nucleus named corneocytes [53]. These keratin-rich cells present a flat shape, which are bound together by lipids such as ceramides, fatty acid and cholesterol; the array of corneocytes and intercellular lipids imparts the SC a “brick and mortar” structure [54]. Right below the SC the Stratum Lucidum is found; this skin layer consists of a single or double layer of partially denuclearized keratinocytes bound together by the eleidin, a lipoprotein that imparts a translucent appearance to this skin layer. It is present only in certain parts of the human body such as feet, palm, and lips [55]. The Stratum Granulosum is located under the Stratum Lucidum, in these layers the keratinocytes own a nucleus and are of granular shape. At this differentiation level, the cells also contain keratohyalin granules, which are rich in the proteins that bind keratin. In this layer the cells secret the interlamellar lipids found in SC; therefore, they are responsible for the skin barrier properties [56]. The under located Stratum Spinosum is constituted by several rows of spine shaped keratinocytes, other cells present in this layer are the melanocytes and the Langherans cells [57]. Melanocytes synthesize melanin and are therefore responsible for skin pigmentation and UV protection, Langherans cells instead contribute to the immune response of the skin [57,58]. On the bottom of the epidermis, the Stratum Basale is composed by a single layer of stem keratinocytes, these cells have large nuclei and high reproduction rate, once produced the stem cell raise to the upper layer gradually differentiating [59]. In the Stratum Basale, together with melanocytes and Langerhans cells, the Merkel cells are found. The latter complies with the role of touch sensors [60].

The Dermal-Epidermal Junction (DEJ) consists of a basement membrane with a laminar structure and binds the dermis and the epidermis together. The DEJ is composed by the Lamina Lucida and the Lamina Densa [61]. The Lamina Lucida is about 50 nm thick, and it has a translucent appearance under transmission electron microscope (TEM) observation; it is mainly composed of laminin, a glycoprotein that interacts with IV type collagen contributing to the binding between epithelial and connective tissues [6,62]. The Lamina Densa is instead thicker (200–300 nm) and mainly composed of IV and VII type collagen together with dermal fibrils and microfibrils [63]. The dermis is located between the DEJ and the subcutaneous tissues (hypoderm); it is vascularized and characterized by a layered structure that can be divided into the papillary and reticular dermis. The Papillary dermis is primarily constituted of connective tissue; it is rich in blood vessels and nerve terminations. It tends to form pegs and ridges with the DEJ; this increases the contact area between dermis and epidermis. The reticular dermis is mainly composed of elastic collagen and oriented fibrous matrices which impart elasticity flexibility and strength to the skin [64]. In this zone the sweat glands are found that contribute to the body thermal regulation, and the hair follicles, which can act as keratinocytes source during the re-epithelialization process. The reticular dermis is attached to subcutaneous tissues without sharp distinctions. The hypodermis is mainly constituted of connective tissue and attaches the skin to the muscular tissues. It is composed of three layers: the top one is rich of fatty substances while the bottom two are vascularized [65]. By observing the overall skin architecture, a complex structure from the biochemical point of view can be easily observed. The alternation of fatty hydrophobic layers and vascularized hydrophilic ones makes it difficult for chemical substances to cross it. Indeed, this natural protection can also act as a shield against therapeutic molecules; therefore, several strategies have been developed in order to make them easily cross the skin barrier [66].

## 3. Skin Interaction with Drugs and Current Challenges in Dermatological Delivery

The significant surface area of the skin has made it an attractive target for drug administration; however, its structural and chemical complexity makes dermatological drug delivery a complex field of study [68]. When dealing with the delivery of active substance to the skin, a significant distinction must be made according to the therapeutic target. Indeed, as previously mentioned, if the therapy aims to act on the skin itself it will be defined as topical administration while if it aims to cross the skin barriers to deliver the drug in the blood vessels, it will be defined as transdermal release [69].

Topical (or dermal) delivery is among the most commonly used administration routes and in general consists in applying the medicament directly on the target tissue and it commonly applies to dermatological and ocular formulations [70,71]. In the dermatological therapies, this local approach is widely used to treat the illnesses that regard the dermis and the epidermis, such tissues being the natural shield that protects the human body from external treat can be affected easily by wounds and infections [72,73]. Common topical release strategies have been used to treat skin diseases such as psoriasis, atopic dermatitis, wounds and skin ulcers [74,75,76]. Such illnesses have been shown to have a substantial impact on patient life, and in the case of severe wounds and ulcers, the patient can be hospitalized and obliged to undergo surgical intervention. For this reason, in recent years, many research efforts have been made in order to allow a more efficient and continuous topical delivery of active substances [77]. Indeed, in the case of chronic wounds, a continuous, prolonged and controlled release of active substances was identified as a smart strategy to stop the advancement of the infection [78]. In addition, the development of biomedical devices that provide a substrate for the re-growth of the skin tissue while topically releasing drug is arising from significant interest [79].

**Figure 2:**
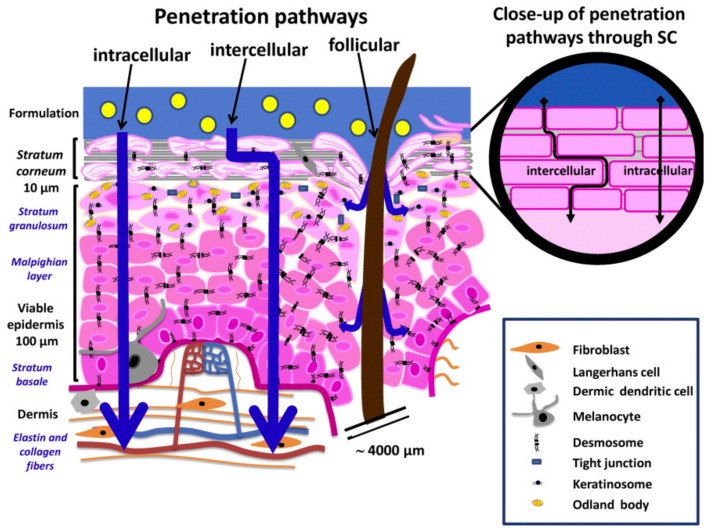
Transdermal transport and penetration pathway (reproduced from Bolzinger et al. (2012) [87], with permission from Elsevier).

Transdermal release involves the penetration by the drug of the skin layers in order to reach the vascularized regions on the epidermis and operate a systemic effect [80]. In Figure 2, the phenomena acting in transdermal release are sketched. For a substance to reach the dermis, three penetration pathways are commonly identified [81,82]. The intercellular route involves the passage around the corneocytes, and the molecules diffuse in the lipid substances that hold the terminally differentiate keratinocytes together, thus such route is generally preferred by lipophilic substances [83]. Oppositely, the intracellular pathway is preferred by hydrophilic substances that cannot diffuse easily in the lipid extracellular network of the SC. This route involves the penetration of the drug through the corneocytes cells [84]. A third path, defined as annexial (or follicular) route, consists of the passage of the drug through the skin annexes such as hair follicles or sweat glands [85]. Notwithstanding that the annexial route allows easy penetration of different drugs across the epidermis, it must be taken into account that skin annexes represent only a small percentage of the overall skin surface and therefore most of the mass transfer occurring in the skin penetration phenomenon would exploit the intra- and extracellular pathways [86].

The percutaneous absorption of a drug can be regarded as a mass transfer phenomenon that involves several steps whose kinetics is mainly dominated by diffusion [88,89]. The first step consists of the entry and penetration of the drug into the SC. Secondly, the drug must partition from the SC into the viable aqueous epidermis reaching the Stratum Basale. The drug must then diffuse through the Stratum Basale in order to reach and permeate the Dermal-epidermal junction. Once it has reached the dermis, the drug must be absorbed by the blood vessels in order to finally enter the systemic circulation [90,91]. The complexity of transdermal penetration lies in the fact that it requires alternate diffusion through different layers and permeation across the boundaries that separate them. Each layer (and boundary) has its own different chemical composition and therefore different resistance to mass transfer. Since a limited diffusion in one single skin layer may hinder the overall penetration process, the amount of active substances easily deliverable transdermally is quite reduced [92]. Common rules found in literature state that in order to cross the SC easily, a molecule should own lipophilic character and small size, i.e., lower than 500 Da; the latter condition is widely known as the 500 Daltons rule [93]. However, lipophilic molecules have also been recently considered to have not optimal penetration capability; indeed, according to some authors, they can easily cross the SC but thereafter cannot diffuse in the vascularized layers, and therefore they tend to accumulate in the SC [94]. A further point to consider is the huge variability of the skin in terms of thickness, chemical composition, and biological activity. Such parameters tend to change in different individuals, the factors that influence the skin physiology include but are not limited to age, sex, phototype and health practices of the patient [95]. Moreover, the relative properties of the individual skin layers can also change in different parts of a single body. For these reasons, the transdermal penetration of a drug is an overall complex phenomenon [96]. Finally, some authors also recently pointed out how skin health status changes the transdermal delivery kinetics [97,98].

Despite the complexity of the transdermal administration route, its potential advantages have driven the scientific community to develop innovative technologies in order to enhance skin penetration by active substances. The physical transdermal penetration strategies consist of applying external stimuli such as electric current or ultrasound to help the drug to cross the epidermal layer [17,99]. The application of low electric current is defined as iontophoresis [100] while the application of ultrasounds (US) is called sonophoresis [101]. Another physical enhancement technology is the Microneedles (MN) one; this technology employs micrometric size needles to pierce the epidermis and deliver the drug directly in the vascularized layers [102,103]. Chemical enhancement instead consists of adjusting the drug formulation by adding appropriate chemical substances that cause the swelling of skin barrier [104]. A further skin penetration enhancement strategy consists of the incorporation of the active substance into nanocarriers; such devices can be designed in order to possess physical and chemical properties that allow them to drive the drug across the skin barrier to reach the systemic circulation without affecting the outer skin integrity [105,106]. Moreover, it is possible to combine some pharmaceutical nanocarriers with textile material in order to produce wearable drug delivery devices. The drug encapsulation is, in fact, one of the most promising technological advancements in the pharmaceutical fields, and for this reason, a deeper discussion is dedicated to it and its application to transdermal drug delivery [107].

## 4. Pharmaceutical Nanocarriers for Dermatological Applications

Pharmaceutical nanocarriers are a nanoscale technology that has been developing over the last decades, given the necessity of administering pharmaceutically active substances most effectively. The encapsulation process is the technique to incorporate active molecules inside nanostructured materials with monolithic or core-shell structure [108]. This process can indeed improve the performances of the active material under many points of views. Firstly, the nanomaterial can protect the drug from environmental factors that alter its biological activity [107,109]. Secondly, the encapsulation process allows to control the structure of the core material in order to regulate its interactions with the environment: as an example, such a technique is commonly used to enhance biostability and bioavailability of poorly water-soluble drugs in the blood stream [110]. Lastly, encapsulation allows releasing the drug in a prolonged and controlled manner providing an effective therapeutic dosage while minimizing sides effects [111]. The most advanced encapsulation technologies can release the drug only if required conditions are verified, making the system stimuli responsive. The encapsulation process is employed to obtain different products and pharmaceutical dosage forms. These formulations are classified according to their chemical nature, structure, morphology, and assemblies. For dermal and transdermal applications, the most studied carriers are micro- and nanoparticles, either polymeric and lipidic, cyclodextrin-based, hydrogels and liposomes [112,113].

Particulate systems are among the most studied carriers; they are classified according to their average size (microparticles, nanoparticles), shell material (organic, inorganic, hybrid) and their morphology and structure (spheres, capsules and bubbles) [114]. In dermatology, the nanosized particles present a wider application range, given their higher capability of penetrating the skin barrier both by intra/extra cellular route and by annexial one. In fact, given the submicronic size of the skin annexes, nanoparticles can pass through them. On the other hand, microparticles own a size that hinders their penetration and are mostly applied in topical skin therapy [66,115]. Concerning the shell nature, mostly polymeric materials are used. This trend is due to the large availability of biocompatible and biodegradable polymers that allows sustained release of the drug by diffusion/erosion of the polymer matrix [116]. Moreover, ceramic materials, such as zinc oxide, can be also used for drug delivery, combining the therapeutic action of the drug with the functional properties of the shell such as antimicrobial or UV light protection [117,118]. According to the structure, polymeric nanocarriers are commonly divided into nanospheres, characterized by a solid structure in which the drug is dispersed in the polymer matrix, nanocapsules, which own a defined core in a liquid state and shell structure, and nanobubbles, spherical core/shell structures filled by a gas or vaporizable compounds [114]. Several authors proposed the application of nanospheres to enhance drug penetration, among them Lalloz et al. [119] deeply studied the effect of the particles’ surface charge on skin penetration. In their work, vitamin D (logP = 7.5) was incorporated in purposely synthesized block copolymers by employing flash nanoprecipitation, a productive nanoparticle production technique (a technique that has been well-described, for example by Lavino et al. [120]). Permeation studies, conducted both on healthy and damaged pig skin, showed a limited effect of the nanoparticle surface charge on the healthy skin while particle polarity affected the release kinetics in the case of damaged skin tissue [119]. By employing a similar particle production methodology, our research group designed poly-ε-caprolactone (PCL) nanoparticles for the delivery of the strongly hydrophilic caffeine molecule (logP = −0.07). The results proved that selection of different solvent for the drug molecule during precipitation process could affect drug partition between the core and the surface of the sphere effecting the release kinetics through the skin annexes [121,122].

Nanocapsules usually present a well-defined core and shell structure. Such devices can either penetrate SC as whole or disrupt and allow the oily core to diffuse in the epidermis. In the work of Pereira [123] adapalene (logP = 8.5) loaded PCL nanocapsules were produced and topically applied: the high hydrophobicity of both carrier and drug facilitated diffusion in the SC but no further penetration was observed, making the system suitable for topical administration. Marto et al. [70,71,72,73,74,75,76,77,78,79,80,81,82,83,84,85,86,87,88,89,90,91,92,93,94,95,96,97,98,99,100,101,102,103,104,105,106,107,108,109,110,111,112,113,114,115,116,117,118,119,120,121,122,123,124] incorporated an anti-inflammatory drug in starch-shelled nanoparticles by emulsion solvent evaporation method. The potential effectiveness of such topical formulation was evaluated both in vitro and in vivo on rats showing an improved efficacy compared to commercial cream formulation. In the study of Mathes [125], the relative performances of both nanospheres and capsules were compared to target hair follicles with glucocorticoids. The particles were prepared by nanoprecipitation solvent evaporation methods, marked with fluorescent dyes and tested both on pig and human skin. This study evidenced how improved targeting of the hair follicles resulted in the drug to be delivered by annexial transport rather than SC permeation. Among the different employed carriers, the nanocapsules displayed a more marked follicular targeting than the nanospheres.

Polymer shelled nanobubbles (NBs) and nanodroplets (NDs) represent another nanotechnology exploited for cutaneous administration of active molecules. They are versatile multifunctional core-shell nanocarriers designed for the delivery of drugs, gases and genes [126]. The structural flexibility of these core-shell systems allows efficient incorporation of several active molecules with high payload. They have been proposed for local delivery of antimicrobial drugs for treatment of cutaneous infectious diseases. Rifampicin-indocyanine green-loaded perfluorocarbon (PFC) nanodroplets were developed as combined photo-chemo-probiotic therapeutics for the treatment of dermatoses caused by P. acnes infection [127]. The combination of vancomycin-loaded NBs with ultrasound was studied to enhance drug penetration through skin by sonophoresis and trigger drug release at the site of infection [128]. In vitro permeation studies with porcine skin showed that the amount of vancomycin accumulated in the skin after US application combined with NBs was greater than the minimal inhibitory concentration (MIC) value, whereas the passive transport through the skin of the free drug was negligible [128]. The feasibility of the use of NB system combined with US was also exploited to design a therapeutic tool to topically treat hypoxia-associated dermal pathologies and promote wound healing process. They showed effective oxygen storing capability and ability to release oxygen to cutaneous tissues both in vitro and in vivo [129,130,131,132,133]. Skin oxygenation of mice topically treated with oxygen-loaded NDs was monitored by visualizing the subcutaneous levels of oxy-hemoglobin and deoxy-hemoglobin through photoacoustic imaging and an increase in the tissue oxygenation with a prolonged oxygen release over time was observed [134].

Cyclodextrins (CDs) are oligosaccharides chemically constituted by glucose rings arranged in a toroidal structure [135]. Common CDs are formed by 6–8 glucose units; moreover, recent discoveries proved the possibility of synthesizing cyclodextrins composed of 3 or 4 glucose units [136]. CDs have been widely exploited in pharmaceutical sciences due to their capability of including active substances in their cavity improving drug dispersibility and bioactivity [137,138]. To improve their drug loading content, several CDs-based supramolecular assemblies have been proposed and synthesized. Nanosponges (NS) are solid nanoparticles consisting of cross-linked cyclodextrins nanostructured within a three-dimensional network [139]. Thanks to their porous nanostructure they are capable of encapsulating hydrophilic as well as hydrophobic molecules, providing sustained and controlled release, improving solubility, stability, permeation and bioavailability [140]. Nanosponges can be incorporated in topical formulations, improving the retention onto the skin of incapsulated drugs. Pyromellitic nanosponges have been proposed as promising multifunctional co-ingredients in topical monophasic and biphasic formulations. Skin permeation studies showed that NS in gels and cream-gels containing diclofenac significantly modulate drug transport through the skin, increasing its amount in stratum corneum and viable epidermis [141]. The same type of NS was exploited for the delivery of imiquimod (IMQ), an immune response modifier. A hydrogel containing imiquimod-loaded NS was formulated to control the scarring process for the treatment of aberrant wounds. Loaded NS could act as a drug reservoir, able to slowly deliver IMQ through the skin, favoring dermal accumulation [142,143]. Permeation studies carried out on porcine skin and rabbit mucosa revealed an enhanced permeation rate of resveratrol loaded in nanosponges compared to free drug [144]. Nanosponge-based hydrogels have been also proposed for the treatment of skin infections. They were designed as alternative carriers for targeting econazole nitrate (EN) to the skin to contrast fungal infection with minimal side-effects [145]. Lemongrass oil-loaded ethyl cellulose nanosponges were developed, showing an enhanced antifungal effect and decreased skin irritation [146].

Liposomes are vesicle-based carriers; they are typically constituted by a phospholipidic bilayer with an aqueous core. Having an amphiphilic structure, liposomes can entrap either hydrophilic or hydrophobic molecules in the aqueous core and in the lipid membrane, respectively. Marhauser et al. [147] have shown that liposomes act as efficient carriers enhancing transdermal drug release. Their approach consisted of preparing fluorescent labeled liposomes and testing their penetration through porcine skin using Franz Cells. The evaluation of the skin penetration was performed both by tape stripping of stratum corneum layer and by horizontal section of the skin membrane followed by fluorescent microscopy observation. Deformable liposomes or transferosomes have been designed to enhance the penetration behavior of traditional liposomes. The high elasticity of these liposomes allows them to squeeze and penetrate the skin by different pathways [112,148]. Hong et al. [149] correlated the effectiveness of the skin transport of liposomal formulations to their mechanical properties. Several formulations with various surfactants were prepared and then their elasticity was measured, and the transdermal release was studied on a porcine skin model.

More recently, other vesicular nanosystems such as niosomes, ethosomes, transethosomes have been studied to enhance the transdermal drug delivery [150]. Ethosomes are novel lipid vesicular nanocarriers composed of phospholipid and a high percentage of ethanol, while transethosomes are the new generation of ethosomal systems containing the basic components of classical ethosomes and an additional compound, such as a penetration enhancer or an edge activator (surfactant) in their composition. They can improve the penetration through stratum corneum by increasing the fluidity of cell membrane lipids [151,152,153,154]. Hydrogels are systems constituted by polymeric chains dispersed in water whose content can constitute up the 99% of the system weight. They are characterized by the presence of several hydrophilic groups in the chains of the dispersed polymer and have a significant efficiency in the encapsulation and the delivery of hydrophilic substances; their high content of water also makes them a suitable material for wound and burn recovery [155]. Jeong and co-workers proposed a hydrogel-based transdermal delivery system obtained by crosslinking carboxymethyl chitosan with hydroxyethyl acrylate via a free radical polymerization mechanism [156]. The synthesized hydrogel network was exploited to load nobiletin with an encapsulation yield of 42%. The hydrogel was proved to be biocompatible with skin tissue by an in vitro cytotoxicity test on keratinocytes cells culture. The skin penetration test on micro pig dorsal skin showed that most of the incorporated drug was released. Moreover, the different hydrogel formulations lead to a higher or lower transdermal penetration for dermal retention, proving that the designed system allowed to achieve the drug delivery in the desired therapeutic target. Taktak et al. [157] used a tri-block co-polymer to produce hydrogels loaded with paclitaxel and heparin for cancer therapy. The formulation shown that can be effective in a combined cancer treatment in vitro and in vivo.

Ceramic materials are also exploited for encapsulation and delivery of active molecules. Indeed, these materials combine the therapeutic effect of the drug with the intrinsic biological activity of the glass [158]. Ceramic materials such as silica, zinca and titania particles are commonly used in the dermatological application since their cations exert antimicrobial activity, and their morphology and surface properties may be tuned to enhance their bio-affinity [159,160,161]. Anirudhan and Nair succeeded in the production of drug loaded mesoporous silica particles with a size range of 200–300 nm [162]. The surface of these particles was grafted with oligomeric chains that acted as thermally and ultrasound-activated gates for drug-controlled release. Biocompatibility was assessed by keratinocytes cell culture assay while skin penetration was observed on excised rat skin. Xu et al. [163] proposed a similar responsive system fully based on inorganic materials. The mesoporous silica nanoparticles were loaded with insulin and capped with ZnO quantum dots. Enzymes were integrated in the system in order to catalyze the conversion of glucose to gluconic acid above a certain sugar concentration. The gluconic acid conversion led to local decrease of pH causing the degradation of quantum dots and triggering the insulin release. The system was proved to be effective in modulating the glucose level on rats. 

Table 1 summarizes the described applications of nanocarriers in dermatological application. It is observable how pharmaceutical nanocarriers have been employed for the release of both hydrophilic and hydrophobic substances. The therapeutic target for which the systems were designed are various and include both topical and transdermal formulations. Most of the reviewed work tested the produced formulations onto porcine skin. Such choice can be interpreted considering the easiness in obtaining pig skin and the proximity of the results obtained on this tissue with the human. In some cases, the formulations were tested in vivo. The obtained results are promising in terms of advancement of such technology toward the clinical trial experimentation stage.

## 5. Bio-Functional Textiles

Pharmaceutical carriers are a promising strategy to provide skin delivery both at topical and transdermal levels. Notwithstanding the efficacy of such carrier in penetrating the skin layers, the application of the nanoformulations is not different from the classical ointments and creams. These administration strategies still require multiple applications by the patient, and therefore do not improve its compliance with the therapy. The strategy of immobilizing the nanocarriers onto textile support was proposed in recent years to overcome this drawback. The obtained materials are defined as bio-functional textiles and consist of a combination of pharmaceutical carriers with a conventional textile material such as cotton, wool or man-made fibers. The great potential of bio-functional textiles lies in the possibility of creating a wearable technology out of the pharmaceutical nanocarriers [47,164].

**Figure 3:**
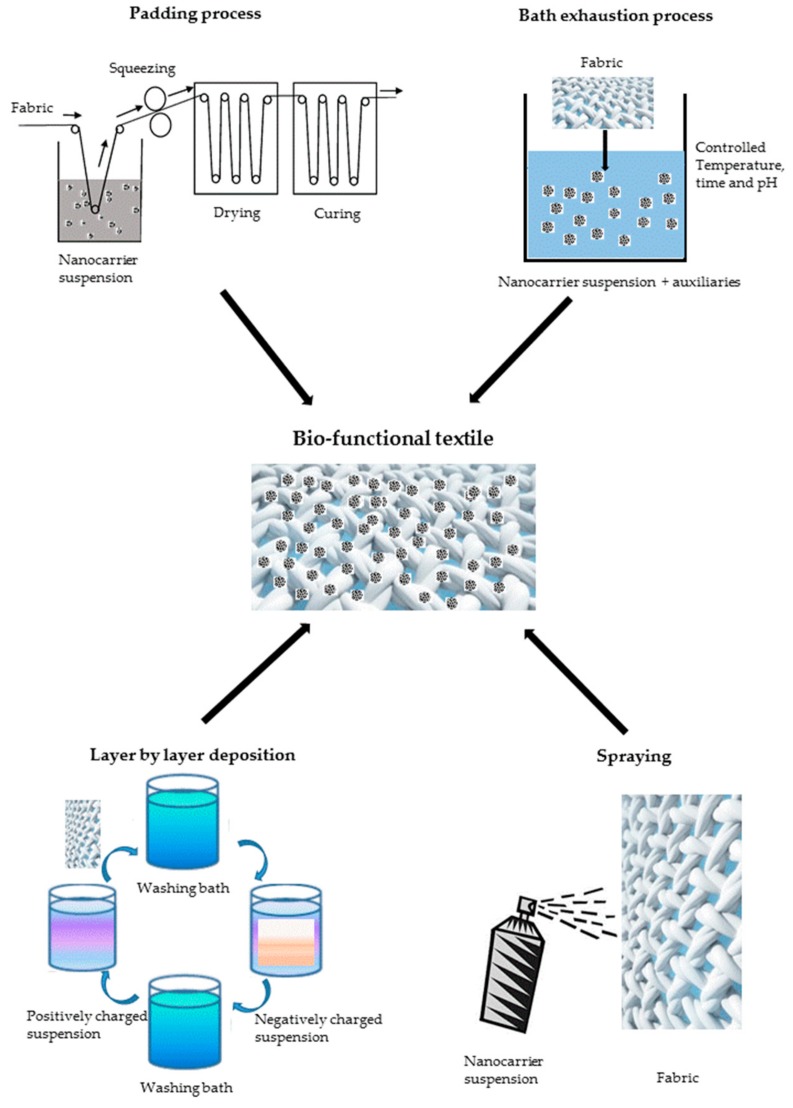
Finishing techniques for bio-functional textile production.

The application of pharmaceutical carriers to a textile generally occurs by mean of a finishing treatment. This is in general the final unit operation of the textile manufacturing chain and aim to impart desired properties to the fabrics both from the aesthetic and technical points of view. In the design of bio-functional garments, the approach is to employ the well-known finishing techniques also for pharmaceutical carriers loading. This approach at the laboratory scale aims to design more easily scalable processes and ease technological transfer. These processes include padding, bath exhaustion, spraying, and layer-by-layer deposition methods, which are sketched in Figure 3. In most cases, drying and curing steps are performed in order to achieve a better fixation of the carriers onto the textile surface [165,166,167]. According to the final applications, the process aims to control the final amount of drugs loaded on the garments; moreover, the fixation treatment is designed in order to tune the interaction strength between the fabric and the carriers. Such interactions play a primary role on the carrier release and the fastness of the treatments [168,169]. The finishing step is realized by means of a wet finishing process for carriers in liquid suspension forms [170]. Bath exhaustion is one of the oldest finishing processes, and it consists of the immersion of the fabric in the finishing liquor. Temperature and pH of the bath are adjusted in order to achieve the best thermodynamic affinity between the fibers and the solution. This technology is batch type; it provides excellent uniformity of the treatment but does also imply some waste of the finishing bath due to the high liquor ratio required [171,172].

Oppositely, padding is a continuous process that allows working with smaller liquor amounts. In this technology, the fabric is quickly immersed in the finishing bath, then squeezed between two rolls, which control the level of wet pick up by applying a given pressure. The padding bath generally contains a binder to promote adhesion of the finishing agent. The padding process requires good affinity with the textiles, and it may not be suitable for inert materials such as polyesters unless preliminary surface activation is performed [173,174,175]. Spray techniques are also used for textile finishing; they can be implemented in process line followed by fixation treatments. Recently, spray-based methodologies such as electrospraying have been proposed to produce the carriers, and therefore they might be a novel approach for a single-step production and finishing process [176,177].

Layer-by-layer (LbL) deposition is a novel finishing technique that arose significant interest in recent years. It consists of alternatively dipping the textile in oppositely charged electrolyte solutions, allowing a precise number of monolayers to deposit on the fiber surface. This technique is widely employed for thin film assembling and is available at pilot scale. All the described finishing techniques can be applied for carrier application onto fabrics [178].

When a cross-linking agent is used, a curing step is usually performed to allow the formation of chemical bonds between the fabric and the carriers, depending on the material end use and the desired design [179]. Curing may require high temperature or UV radiation in order to effectively promote chemical reactions, however, in the context of bio-functional textiles, particular care must be taken in order to avoid drug degradation or carrier deterioration under the curing process conditions [172].

The produced bio-functional textiles are usually studied and tested at laboratory scale to assess their performance. Characterization of the product aims at determining the effectiveness of the finishing technique, which is commonly investigated by spectroscopy-based analysis such as attenuated total reflectance Fourier transform infrared spectroscopy (ATR-FTIR) and morphological observation by scanning or transmission electron microscopy (SEM, TEM). Moreover, durability of the treatment is usually tested in terms of washing fastness. This test is commonly conducted by washing the textile material several times, according to textile norms and standards, and repeating the ATR-FTIR and SEM analysis to qualify and quantify the residual drug on the fabric [41,180,181]. Pharmaceutical analyses are instead aimed to evaluate the potential effect of the product on the living body. They usually are designed according to the therapeutic target of the bio-functional textile. The transdermal release is usually evaluated by the Franz diffusion cell method, which is the test recommended by the pharmacopeia for dermal and transdermal formulations. The Franz cell consists of a vessel divided into a donor and acceptor compartments; the acceptor one is filled with a blood mimicking solution, while the formulation is placed in the donor one. The two compartments are separated by a membrane, which mimics the skin; it can be constituted by artificial or real skin obtained from rat, pigs, or human donors [182,183]. The kind of membrane used in the test is the main variable in determining the overall results, and recently many attentions have been paid on it. The group of Martì recently proposed the use of novel artificial membranes enriched with SC mimicking lanolin which could combine the desirable low variability of artificial membranes while maintaining the diversified layer composition typical of real skin [184]. A complete review of the physical and chemical properties of excised skin was done by Todo, who also analyzed the possible effect on a transdermal penetration test [185]. Cell cultures are also a widely used tool to assess bio-functional textile in in-vitro performance. They are commonly used to either test the product compatibility with healthy skin cells or to evaluate the capability of the textile to protect the person from harmful microorganisms. In the first case, the cell culture is made of skin keratinocytes or corneocytes, cell viability may be measured by methylthiazolyldiphenyl-tetrazolium bromide (MTT) or Lactate dehydrogenase (LDH) assays [186,187]. In the second case, the microbial growth inhibition is conducted on model bacteria, such as the gram-positive *Staphilococcus Aureus* or the gram-negative *Escherichia Coli*, or fungi such as *Candida Albicans*. The antimicrobial tests are realized according to several standard protocols, and many authors repeated them after washing the textile in order to assess the antimicrobial finishing fastness [172,186,188]. Lastly, non-invasive in-vivo methodologies consist in applying the textile on the skin of human volunteers in the form of a patch test and subsequently obtain data on the skin condition by different methods such as tape striping or transepidermal water loss measurement (TEWL) [189].

Our research group proposed the finishing of cotton fabrics with polycaprolactone (PCL) nanospheres. The particles were produced by Flash nanoprecipitation method exploiting the confined impinging jet reactor. Several drugs were loaded in the particles and thereafter applied to cotton by simple imbibition in order to enhance the detachment of the nanospheres from the textile [190]. The garments were tested according to the effect of the loaded substances. In the case of menthol-loaded textiles, aimed to provide topical refreshment feeling to the wearer, they were applied on a set of volunteers in a double blind protocol, the subjects were interviewed and skin parameter measured, proving that the functionalized cotton can provide refreshment without causing skin irritation [189]. The study conducted on melatonin-loaded fabrics was aimed at promoting drug crossing through the skin membrane and reaching the systemic circulation. Therefore, the functionalized fabric was mounted on a Franz cell, and this study proved an effective release of the drug through the skin mimicking membrane [164].

Micro- and nanocapsules are among the most employed carriers employed in textile finishing for a wide range of applications [107,191]. In the context of bio-functional garments, various kinds of core substances and shell materials can be combined in the capsule formation in order to impart health beneficial properties. In the work of Yang et al. [192], a spray drying method was employed to incorporate vanillin in a chitosan shell, the capsules were grafted on cotton fabrics by a bath exhaustion protocol in which citric acid was added to promote crosslinking. The obtained textile exhibited high washing fastness due to the chemical bonds between chitosan shell and the cellulose fabric. Ghayempour et al. [193] have recently incorporated the hydrophilic chamomile extracts via a double emulsion method, the obtained capsules where mixed with an acrylic resin and applied to cotton fabric, the resin was crosslinked by UV curing. Such an approach led to slow release and high washing fastness due to presence of the capsules and the resins hindering the extract diffusion. Furtherly, the hindered diffusion did not compromise the antimicrobial activity of the extracts retained throughout several washing procedures. According to Martì et al. [168], the application of the capsules by resin finishing may cause considerable alteration on the transdermal release kinetics. The transdermal release occurs by diffusion phenomena, the driving forces depending on the concentration gradient, which is affected by the amount of drug contained in the fabric. For this reason, this study focuses on evaluating the effective drug loading of the fabric upon treatment with microcapsules and liposomes. The application was performed by padding process onto cotton, polyamide (PA), polyacrylic (PAC) and polyester (PES). The substance was extracted both by soxhlet extraction and ultrasound bath and quantified by UV-vis spectroscopy and HPLC. The work proved that in the carrier loading onto different fabrics the great role is played by surface interactions and chemical affinities between the carrier shell and the fiber [168]. The same research group intensely studied the transdermal release from fabrics functionalized with liposomes. Firstly, they investigated the release of caffeine from liposomes attached onto cotton surface by comparing the results of Franz Cell experiments performed with synthetic membrane and porcine skin [194]. In the following test, the bio-functional textiles were also functionalized with liposomes produced with internal wool lipids in place of phosphatidylcholine. For these material tests, a non-invasive in vivo test methodology was also introduced [195].

Cyclodextrins (CDs)-based finishes have been widely exploited in recent years. Martel’s group proposed a methodology that allows the chemical modification of cyclodextrins to impart positive and negative surface charges [196]. After cationization or anionization, the CDs were loaded with 4-tert-butylbenzoic acid (TBBA) and deposited on a polyester substrate by LbL deposition. The polyester substrate was preliminarily activated by plasma treatment, and after deposition, the material underwent thermal curing in order to promote the formation of chemical bonds between the oppositely charged cyclodextrins layers. A controlled release profile was assessed by a flow through cell apparatus and cell culture proved both the non-cytotoxicity and the antimicrobial activity [188]. Mihailiasa et al. [197] synthesized cyclodextrin nanosponges by chemical crosslinking. The crosslinking was proven to be useful to improve drug loading. Moreover, the loaded nanosponges were applied on cotton fabrics by bath exhaustion process and the treatment exhibited fastness for two washing cycles. The release test on Franz cell showed zero-order release kinetics. Maestà Bezerra et al. [198] produced similar crosslinked cyclodextrin systems which were employed for the incorporation of citronella oil. The CDs were applied on wool fabrics by padding method, the overall system presented an anomalous release kinetics according to the Korsmeyer and Peppas’s model.

Textile finishing with inorganic particles was proposed by Hassabo et al. [199], who incorporated various topical medicaments such as diclofenac and linoleic acid into silica nanoparticles. The particles were synthetized with the Staber method using TEOS as precursor and applied to cotton by a spray-based methodology. The release test proved the release of the active substance in dermal pH condition and the antimicrobial activity against several species. A combination of organic and inorganic particles was proposed by Perelshtein et al. [200], which employed sonodynamic deposition method. This approach allowed the direct assembly of the particles onto the cotton surface eliminating, therefore, the need for a finishing treatment. The combination of zinc oxide (ZnO) and chitosan significantly improved the antimicrobial activity compared to the single agents and the use of ZnO particles also imparted UV protection properties to the garment. The use of hydrogels in the context of bio-functional textile preparation was proven to be an effective strategy to provide drug administration and moisture management simultaneously [155]. The work of Hui et al. [186,187] exploited an emulsion cross link process to produce chitosan hydrogels loaded with Chinese herbal medicines. The obtained carriers were applied to cotton fabrics by exploiting the pad-dry-cure method with a chemical binder. The MTT and LDH tests confirmed the non-cytotoxicity of the material while the release test displayed a sustained drug administration for an entire week. The system was considered suitable for treatments of atopic dermatitis and other topical applications due to micrometric size of the carriers, which prevented transdermal permeation.

Petrusic et al. [201] proposed micro-hydrogel for the design of stimuli response drug releasing textiles. The hydrogel was constituted of a thermo-responsive polymer (Poly(N-isopropylacrylamide)), which undergoes significant swelling in the range of skin temperatures. Hydrogels were prepared from an inverse suspension polymerization method. The Franz cell experiments were conducted at different temperatures and shown that the thermosensitive hydrogels are an effective strategy to control the amount of procaine released [201,202]. Table 2 reports the described characteristics and application of bio-functional textiles. It can be observed that cotton fabrics are among the most exploited textile material for this specific application. Such observation can be easily explained by considering comfort and biocompatibility of cellulosic material such as cotton. Furtherly, cotton is extremely wettable and therefore surface functionalization can be easily performed by wet finishing treatments. The applications of bio-functionalized textile materials are of different kinds: in all the cases, however, an active substance with specific therapeutic or cosmetic properties is released from the textile system. The finishing treatments that have been employed for bio-functionalization are diverse; the choice of each author made on the basis the final application. The objective is selecting the functionalization process that will promote the right interaction among the carriers and the fibers in order to achieve the desired properties in terms of fastness and controlled release. The opportunity of adapting the textile finishing processes to different pharmaceutical carriers is promising in terms of process scalability.

## 6. Bio-Functional Textiles and Other Dermatological Delivery Technologies

To better understand the significance and the potential of the bio-functional textile approach, other technologies aimed to deliver drugs to and across the skin are analyzed and discussed.

Conceptually similar to bio-functional fabrics, electrospun nanofibers have gained significant interest in the context of biomedical research over the last years [203,204]. Such materials consist of fiber mats with a diameter below the micrometer. These nanofibers are obtained through the electrospinning process, consisting of spinning a viscous polymer solution in a high voltage electrical field. The fibers are collected on a metal plate and during the way from the tip to the collector, the solvent evaporates allowing a dry fiber mat to be collected [205,206]. The process is very versatile and allows spinning of different polymers. Therefore, in the context of biomedical applications, several bio compatible polymers, such as PCL, chitosan, silk fibroin, keratin, alginate, etc. have been electrospun. Moreover, drugs or drug loaded nanocarriers can be introduced in the viscous solution, therefore spinning drug eluting nanofibers [207,208]. In the frame of dermal therapy, this approach is very promising for wound recovery application. Indeed, wound therapy requires both the promotion of the cellular regrowth and the administration of drugs to fight bacterial infections. Drug-loaded nanofibers can elute drugs on the damaged tissue to prevent bacterial growth. Moreover, their structure was found to be very similar to the extracellular matrix from the morphological point of view; therefore, this material can promote healthy cells regrowth [208,209].

Microneedles (MN) technology consists of the production of patches equipped with several arrays of needle of small size. The aim of MNs is to inject the drug inside the deeper vascularized skin layers by creating micron sized holes in the epidermis. Thus, such technology overcomes the issues of permeation trough the epidermal layer. MN can load drug in the patch, in the tip of needle or just on its surface [102,210]. The material used to fabricate the needle can be a metallic, ceramic or polymeric compound [211]. In the recent years the polymeric MNs have aroused the greatest interest.

Indeed, they have been proven to be cost effective due to the lower cost of polymeric material and for the simplicity of the production process. Furthermore, in some cases the polymer itself is bioabsorbable and degrades inside the dermis. To achieve these effects, the polymer must be biodegraded in dermal physiological conditions, for this reason polymer like Polylactic acid (PLA), Polyvinylpyrrolidone (PVP) and chitosan are usually preferred [212]. The possibility of delivering the drug in vascularized dermis avoiding a fraction of the drug to be blocked in SC made MNs among the most effective transdermal technology. On the other hand, some concerns are usually raised by the high invasiveness of such a technique which indeed pierces and holes the skin and may cause irritation. For these reasons, MNs may be considered an effective approach to administer drugs such as insulin and chemotherapeutics. These drugs are generally quite expensive, so their administration must be maximized. Moreover, their common administration route is the parenteral one and therefore MN is a novel and less invasive administration route [213,214]. Other skin penetration technologies imply the use of external stimuli in order to promote the permeation of the therapeutic agent in the epidermis [215]. Sonophoresis exploits ultrasounds to enhance the permeation by disrupting the Stratum Corneum [216]. Sonophoresis can be operated either at therapeutic frequencies (1–3 MHz) or at low frequencies (20–100 kHz). The mechanism of penetration enhancement by sonophoresis is still to be fully understood. The main proposed effects are the acoustic cavitation of gas microbubbles that causes the disruption of the extracellular lipids in the SC. Moreover, permeation is enhanced by a local gradient of temperature generated by the ultrasound energy, which plays a role in promoting diffusion [216,217]. The employment of low frequency sonophoresis was proved to be efficient in promoting the permeation of high MW molecules or small hydrophilic ones [101,218]. However, the effects of penetration enhancement and the pharmacokinetics upon the ultrasound treatment significantly vary on the kind of drug under investigation [219]. The enhancement of skin penetration obtained by applying an electrical stimulus is defined as iontophoresis. This technique employs low electrical currents (below 0.5 mA/cm^2^) to promote the active molecules permeation through the epidermis. Concerning the electrically promoted transdermal transport mechanism, two main phenomena have been identified. The electrophoretic mechanism is mainly based on the repulsion of the drug from the electrode induced by the applied current, the drug is in this way pushed to cross the skin barrier [17]. The electroosmotic mechanism works in parallel with the electrophoretic one and can either promote or hinder drug permeation. In fact, considering the epidermis as a porous membrane in which the charges are balanced, the application of an electrical current would cause an ion flux across the membrane. Therefore, the drug transport based on iontophoresis will be strongly influenced by the polarity and surface charge of the species to be delivered and on the pH of the skin [220]. The penetration route enhanced by iontophoresis is mostly the annexial one. Indeed, iontophoresis has been widely studied to promote penetration of different kind of drugs or the transport of surface charged polymeric nanocarriers [221]. In order to apply the iontophoresis technique, the drug must be charged and satisfy certain polarity requirements [100]. To critically compare the mentioned technologies, several prospective should be considered. In Table 3 the main aspect related to transdermal technologies are compared.

Concerning the kind of drugs that can be delivered by each system ionto- and sonophoresis present strict limitations compared to MN and bio-functional textiles. In fact, once the active substance is incorporated in the carriers and in the MN the release kinetics is less affected by the chemical structure of the therapeutic molecule. The release phenomena occurring are also of different nature. In MNs the permeation of the epidermis is bypassed, making the release from this technology more effective, while in bio-functional textiles, the permeation mechanism is mostly dominated by passive diffusion. A textile-based delivery system could potentially be applied to all the drugs that are deliverable by a nanocarrier system. Therefore, it can be stated that bio-functional textiles own the potential of administering numerous kinds of drugs. Concerning the control of delivered dosage, it can be stated that its accuracy is strictly related to the complexity of the release mechanism. MNs indeed offer the best dosage control since the needle system penetrates the epidermis and dissolves in the vascularized dermis releasing the total amount of drug loaded in the needle. Ionto- and sonophoresis require, instead, the drug to permeate all the layers of the skin undergoing the already discussed slowing-down or accumulation phenomena which makes the control of the dosage less accurate compared to MNs.

A further level of complexity is found in the textile systems. Here the drug is generally encapsulated and therefore the permeation phenomenon is a combination of the carrier penetration together with the release of the drug from the carrier, two mechanisms that have different characteristics times and potentially overlap. Moreover, the kinetics of release of the carriers from the textiles plays a critical role in the overall drug administration process. For this reason, the overall release of drugs from a bio-functional textile is more complicated with respect to the other systems and is yet to be fully understood in order to achieve an effective control of the dosage. Concerning the invasiveness of the systems, MNs require the outer layers of the skin to be perforated. Furthermore, if the polymer swells inside the epidermis, the needles enlarge, causing the micropuncture to increase in size. Ionto- and sonophoresis require external stimuli that may perhaps damage skin integrity. Moreover, proper devices should be used to apply electricity and ultrasounds. Therefore, ionto- and sonophoresis are more difficult to be operated by the patient without medical supervision. Bio-functional textiles are instead quite simple to be applied since they can be worn as conventional garments or bandages. Moreover, their application has never been reported to cause side effects on the patient’s skin. It can be observed that bio-functional textiles are a promising technology under many points of view. Their main current weakness is the scarce control over the amount and kinetics of drug release related to high complexity of phenomena occurring. However, such limitations may be overcome in the coming years upon the conduction of proper research and studies. Indeed, the research efforts necessary to optimize these systems may be worthwhile, providing their great advantages in terms of biocompatibility, easiness and versatility.

## 7. Regulatory Status

Given the great potential and interest in the development of bio-functional textiles, an overview of the regulatory status of such devices is provided in order to better understand the challenges and opportunities of such materials in reaching the market. The application of nanomaterials in wound care and medical textiles is rapidly increasing. Considering the current regulatory status, the classification of a textile product should consider several aspects. A textile containing active molecules loaded in nanocarriers can be considered either a medical device, a cosmetic or a medicinal product, according to the type of encapsulated molecule and the permeation profile. Moreover, it is worth underlining that a drug incorporated in a medical device must have only an ancillary function, otherwise the product must follow the regulation of medicinal products [222]. In addition, the current European regulatory framework supervising the presence of nanomaterials in healthcare products is complicated, because it is not well-defined and not yet harmonized. Nevertheless, Regulatory Agencies required manufacturers to perform accurate studies for assessing the quality, safety, and efficacy profile of a nanotechnology-based product [223]. In July 2007, the Nanotechnology Task Force of FDA prepared a report presenting an assessment of scientific and regulatory considerations relating to the safety and effectiveness of FDA-regulated products containing nanomaterials. Moreover, the FDA has issued several additional guidance documents to industry in order to provide recommendations for evaluating the application of nanotechnology in FDA-regulated products [224,225,226]. The safety of a product containing nanomaterials is a key factor to be assessed. A risk management strategy is required in order to market nanoparticle-loaded medical devices. In January 2015, the European Scientific Committee on Emerging and Newly Identified Health Risks (SCENIHR) adopted an Opinion on the “Guidance on the determination of potential health effects of nanomaterials used in medical devices”. This Guidance provides information about the safety evaluation of nanomaterials, i.e., the determination of hazards associated with the use of nanomaterials and risk assessment for the use of nanomaterials in medical devices [227]. The ISO guideline (EN ISO 10993) concerning the biological evaluation of medical devices reports the basis for the biological safety tests for textiles. This indicates which risk analyses and test (i.e., cytotoxicity, tissue compatibility, sensitization and irritation potential evaluation) need to be carried out [228]. In addition, there is a stricter classification for devices containing nanomaterials, considering the potential internal exposure to nanoparticles caused by the application of such a product. As far as the FDA is concerned, existing regulations have been adapted for medical devices with nanoparticulate components. A medical device containing nanomaterials should undergo premarket testing and approval under the Premarket Approval Application (PMA), if the inclusion of nanomaterials requires original clinical study data to ensure the safety of the nanoproduct. Indeed, this approval process requires clinical trials and/or other evidence to demonstrate the safety and effectiveness of the device [229,230]. Conversely, the application of nanomaterials in cosmetics is well-defined and regulated. Regulation (CE) No 1223/2009 introduced specific requirements for marketing cosmetic products containing nanomaterials. Responsible persons (i.e., manufacturers, importers or third persons appointed by them) are required to register cosmetic products on the cosmetic products notification portal (CPNP), specifying the presence of nanomaterials in the product, together with their identification and the foreseeable exposure conditions. Moreover, if the European Commission has concerns regarding the safety of a nanomaterial, it may request the scientific committee on consumer safety (SCCS) to perform a risk assessment [231]. FDA provides guidelines to industry on the safety assessment of nanomaterials in cosmetic products. The guidance documents help to identify the potential safety issues of nanomaterials in cosmetic products and develop a framework for evaluating them. In particular, the safety of a cosmetic product containing nanomaterials should be evaluated by analyzing the physicochemical properties and the relevant toxicological endpoints of each ingredient in relation to the expected exposure [232].

## 8. Conclusions

The present review covered the research conducted in the recent year on the application of textiles materials as transdermal delivery devices. The structure of the skin was deeply described from the chemical biological point of view. This allowed us to have a clearer understanding of such a complex organ both as a therapeutic target or as a barrier to be crossed to deliver the drug systemically. The possible interactions of drug with skin were discussed with a focus on the different pathways of transdermal administration. It clearly emerged that transdermal penetration is influenced by several complex phenomena that are hindering its wider application. The application of pharmaceutical nanocarriers was therefore analyzed as a novel strategy to overcome the skin barrier. Indeed, it was discussed how many encapsulation technologies have nowadays proved their efficacy in enhancing skin penetration. Moreover, such technology showed to be quite versatile and able to deliver different kinds of active substances for various applications. Bio-functional textiles were therefore presented as a combination of these carriers with textile fabrics. The different methods to apply the carriers on the fabrics were discussed. It was shown that common textile finishing apparatus are suitable for the carrier application. The literature review of the novel bio-functional textiles showed the possibility of developing garments with beneficial properties to human health. The bio-functional textiles technology was critically compared with other transdermal penetration approaches. It was clearly evidenced that its versatility and compatibility make bio-functional textile technology significantly advantageous. The regulatory analysis evidenced how the commercialization of a bio-functional textile may depend on the product characteristics in terms of incorporated substance and employed nanocarrier. The literature review here conducted provided a broad view on a topic that matches pharmaceutical and healthcare industry with the textiles one. It can be concluded that the emerging technology of bio-functional textiles is a link between these two sectors since it matches the most advanced pharmaceutical technologies with the smart textiles technologies.

## Figures and Tables

**Table 1:** Characteristics and application of nanocarriers in dermatological therapies.

Drug	Hydrophilicity	Carrier	Therapeutic Indication	Experimentation	Ref.
Vitamin D	Hydrophobic	Nanospheres	Supplement administration	Healthy and damaged porcine skin	[119]
Caffeine	Hydrophilic	Nanospheres	Antioxidant and anti-cellulite	Artificial Membrane	[122]
Adapalene and adapsone	Hydrophobic and hydrophilic	Nanocapsules	Dermatitis treatment	Porcine skin	[123]
HNE inhibitor	Hydrophobic	Nanocapsules	Psoriasis	In vitro and in vivo (rats)	[124]
Clobetasol propionate	Hydrophobic	Nanospheres and nanocapsules	Alopecia treatment	Ex vivo pig and human skin	[125]
Vancomycin	Hydrophilic	Nanobubbles	Skin infection	Porcine skin	[128]
Rifampicin	Hydrophobic	Nanobubbles	Acne treatment	In vitro studies	[127]
Imiquimod	Hydrophobic	Nanosponges	Aberrant wounds	Porcine skin	[143]
Resveratrol	Hydrophobic	Nanosponges	Antioxidant	Porcine skin	[144]
Econazole nitrate	Hydrophobic	Nanosponges	Fungal infection	In vitro studies	[145]
Sodium Fluorescein	Hydrophilic	Liposomes	Model system	Porcine skin	[147]
Quercitin	Slightly hydrophilic	Liposomes	Antioxidant	Human excised skin	[149]
Nobiletin	Hydrophobic	Hydrogel	Acne treatment	Porcine skin	[156]
Heparin and Paclitaxel	Hydrophilic and hydrophobic	Hydrogel	Transdermal cancer therapy	In vitro and in vivo	[157]
5-fluroracil	Hydrophilic	Silica nanoparticles	Cancer therapy	Rat skin	[162]
Insulin	Hydrophobic	Silica nanoparticles and ZnO quantum dots	Transdermal diabetes therapy	In vivo in rats	[163]

**Table 2:** Characteristics and application of bio-functional textiles.

Carrier	Active Substance	Textile	Carrier	Finishing Technique	Application	Reference
Poly-ε-caprolactone (PCL) nanospheres	Melatonin	Cotton	PCL nanospheres	Imbibition	Transdermal delivery	[164]
PCL nanospheres	Menthol	Cotton	PCL nanospheres	Imbibition	Thermal regulation	[189,190]
PCL nanospheres	Caffeine	Cotton/Micromodal	PCL nanospheres	Imbibition	Antioxidant activity	[121,122]
Chitosan microcapsules	Vanillin	Cotton	Chitosan microcapsules	Bath Exhaustion/Crosslinking	Antibacterial and aroma release	[192]
Chitosan microcapsules	Chamomile extracts	Cotton	Chitosan microcapsules	Resin finishing/UV curing	Topical antibacterial	[193]
Liposomes and microcapsules	Sunscreen	Cotton, PA, PAC, PES.	Liposomes and microcapsules	Foulard	UV protection	[168]
Liposomes	Sunscreen	Cotton	Liposomes	Bath Exhaustion	UV Protection	[195]
Liposomes	Caffeine	Cotton	Liposomes	Imbibition	Transdermal administration	[194]
Cyclodextrins	4-tert-butylbenzoic acid (TBBA)	polyester (PES)	Cyclodextrins	Layer by layer deposition	Topical infections treatment	[188,196]
Cyclodextrin nanosponges	Melatonin	Cotton	Cyclodextrin nanosponges	Bath Exhaustion	Transdermal release	[197]
Cyclodextrin	Citronella oil	Wool	Cyclodextrin	Padding	Insect repellency	[198]
Silica nanoparticles	Diclofenac	Cotton	Silica nanoparticles	Spray	Topical treatment	[199]
Chitosan hydrogel	Chinese Herbal extract	Cotton	Chitosan hydrogel	Pad-dry curing	Topical treatment	[186,187]

**Table 3:** Comparison among different transdermal technologies.

	Bio-Functional Textiles	Microneedles	Sonophoresis	Iontophoresis
Drug applicability	Drugs deliverable by nanocarrier system	Most of the drugs	Small substances	Charged and polar drugs
Penetration mechanism	Complex: release from textile + transdermal penetration	Simple: direct release in the epidermis	Transdermal penetration + cavitation	Electro osmosis and electrophoresis
Control of dosage	Lower due to complex release	Very Good	Fair	Fair
Patient usability	Simple to wear	Simple patch application	Ultrasound device needed	Electrical current to be applied
Administration required	Few	Few	Several	Several
Possible side effect	None reported	Skin piercing and irritation	Stratum Corneum (SC) disrupted	Surface damages
